# Prognostic value of Notch receptors in postsurgical patients with hepatitis B virus‐related hepatocellular carcinoma

**DOI:** 10.1002/cam4.1077

**Published:** 2017-05-31

**Authors:** Tingdong Yu, Chuangye Han, Guangzhi Zhu, Xiwen Liao, Wei Qin, Chengkun Yang, Zhen Liu, Hao Su, Xiaoguang Liu, Long yu, Zhengtao Liu, Sicong Lu, Zhiwei Chen, Yu Liang, Jianlu Huang, Xue Qin, Ying Gui, Jiaquan Li, Tao Peng

**Affiliations:** ^1^ Department of Hepatobiliary Surgery the First Affiliated Hospital of Guangxi Medical University Nanning 530021 Guangxi China; ^2^ Department of Hepatobiliary Surgery Affiliated Hospital of Guangdong Medical University Zhanjiang 524001 Guangdong China; ^3^ Department of Hepatobiliary and Pancreatic Surgery the First Affiliated Hospital of Zhengzhou University Zhengzhou 450000 Henan China; ^4^ Department of Hepatobiliary Surgery the Third Affiliated Hospital of Guangxi Medical University Nanning 530031 Guangxi China; ^5^ Department of Clinical Laboratory the First Affiliated Hospital of Guangxi Medical University Nanning 530021 Guangxi China; ^6^ Department of Clinical Laboratory Center the First Affiliated Hospital of Guangxi Medical University Nanning 530021 Guangxi China; ^7^ Medical Scientific Research Center Guangxi Medical University Nanning 530021 Guangxi China

**Keywords:** hepatitis B virus, hepatocellular carcinoma, mRNA, notch receptors, prognosis, SNP

## Abstract

Hepatocellular carcinoma (HCC) is one of the most prevalent malignancies and a major cause of cancer involved death worldwide. Prognosis remains poor because of high recurrence rates and lack of effective relapse prevention strategies. Notch pathway plays an important role in tumor progression and metastasis, and it is associated with the prognosis of cancer. A total of 465 hepatitis B virus (HBV)‐related HCC patients who underwent surgery were enrolled. Single nucleotide polymorphisms (SNP) of Notch pathway receptors were genotyped using Sanger DNA sequencing. Kaplan–Meier curves and the Cox proportional hazards regression model were adopted to analyze the association of polymorphisms and mRNA expression with clinical and pathological features, respectively. Four SNPs (rs1043996 in Notch3 and rs422951, rs520692, rs3830041 in Notch4) were significantly associated with overall survival (OS) (*P *=* *0.023, *P *=* *0.042, *P *=* *0.028, and *P *=* *0.001 respectively). Patients carrying the AA genotype in rs1043996 and TT/TC genotypes in rs422951 and rs520692 significantly decreased risks of death, compared to those carrying the AG/GG genotype in rs1043996 and CC genotype in rs422951 and rs520692, respectively. Patients carrying the TT genotype in rs3830041 showed poorer OS, compared with those carrying the TC/CC genotype. A haplotype block (rs422951 was in strong LD with rs520692, *r* ^2^ = 0.843) was identified in Notch4. Notch3 mRNA expression significantly increased in tumor tissue, compared with nontumor normal tissue (*P *<* *0.0001). Moreover, higher expression of Notch3 was associated with poorer OS (HR = 2.11, 95% CI = 1.32–3.37, *P *=* *0.002) and shorter recurrence time of HBV‐related HCC (HR = 1.96, 95% CI = 1.31–2.93, *P *=* *0.001). Our findings collectively indicate that Notch receptors variants (rs1043996 in Notch3 and rs422951, rs520692, rs3830041 in Notch4) are independent predictive targets for OS in HBV‐related HCC patients. Notch3 expression is a potential prognostic biomarker of OS and recurrence‐free survival (RFS) prediction in HBV‐related HCC patients following surgical treatment.

## Introduction

Liver cancer is one of the prevalent malignancy types and has high cancer‐related mortality rate. In 2012, estimated 782,500 new cases were diagnosed with this cancer resulting in approximately 745,500 deaths worldwide. China accounts for ~50% of morbidity and mortality particularly 1. Hepatocellular carcinoma (HCC) is the major histologic type, representing 90% of the total liver cancer cases 2. By 2015, the incidence of HCC had rapidly increased to 446.1 per 10,000 and the mortality rate was up to 422.1 per 10,000 in China 3. Chronic HBV infection contributes to over 50% and 60% of liver cancer cases worldwide 4 and in China alone 5, respectively. Surgical resection is a potentially curative option. However, the prognosis of hepatocellular carcinoma (HCC) after hepatectomy remains poor because of the high recurrence rate and lack of effective adjuvant therapeutic strategies 6. Tumor recurrence after resection leads to complications in more than 70% cases at five years 7. Therefore, novel prognostic and predictive markers and individual treatments are urgently required to improve the outcome of HCC.

The Notch pathway is a highly conserved signaling pathway that contains four transmembrane receptors (Notch1‐4) and five ligands (Jagged1, Jagged2, and Delta‐like 1, 3, and 4) in humans 8. This pathway not only plays a vital role in cellular functions, including proliferation, apoptosis, cell fate, and differentiation 9, but also tumor angiogenesis and metastasis 10. Accumulating evidence has established an association of Notch expression with biological behavior and prognosis of cancers. High Notch1 expression has correlation with progression and poor prognosis of various tumors, including breast cancer 11, ovarian cancer 12, nonsmall cell lung cancer 13 and colorectal cancer 14. Another study suggests that Notch3 overexpression is correlated with recurrence of ovarian cancer 15. However, Yash R. Somnay, et al. reveal that higher Notch3 expression in thyroid cancer is associated with increased overall survival (OS) compared with lower expression group 16.

Single nucleotide polymorphisms (SNPs) have been identified as potential biomarkers for disease research, including association with pathogenesis and prognosis of cancer. Several studies have demonstrated that genetic polymorphisms are linked with the prevalence and clinical outcomes of HCC. Genome‐wide association studies (GWAS) clearly indicate that SNPs increase susceptibility to HCC in patients with HBV infection 17 and HCV infection 18. SNPs play a potential role in the development of HCC and prognosis of HCC patients with HBV infection 19. While several SNPs have been linked to clinical outcomes or prognosis of HCC, no studies to date have demonstrated the potential association between SNPs of Notch receptors and HCC progression and prognosis.

In this study, we investigated the association of polymorphisms and mRNA expression of Notch receptors with clinical outcomes in HBV‐related HCC, with the aim of identifying effective prognostic biomarkers and therapeutic targets for patients receiving hepatectomy.

## Materials and Methods

### Study population and data collection

A total of 465 HBV‐related HCC patients were consecutively recruited between 2005 and 2013 at The First Affiliated Hospital of Guangxi Medical University (Guangxi, China). The enrollment criteria included patients with positive hepatitis B surface antigen (HBsAg) and those receiving surgical resection to remove primary tumor histopathologically confirmed as HCC after surgery. Patients with HCV infection, OS of less than 3 months and other cancers were excluded.

Clinicopathological data were collected from medical records and pathological reports (details in Table 1). The clinical tumor stage was classified based on the Barcelona Clinic Liver Cancer (BCLC) staging system 20.Child‐Pugh class was defined as reported previously 21. Smoking status, drinking status, and criteria of radical resection used the criterion of previous report 22.

**Table 1:** Patient characteristics and clinical outcome analysis

Variables	Patients (*n* = 465)	MST (months)	OS		MRT (months)	RFS	
			HR* (95% CI)	*P* *		HR* (95% CI)	*P* *
Age (year)							
≤45	236	65	Ref.		4	Ref.	
>45	229	58	0.88 (0.67–1.15)	0.353	5	0.86 (0.64–1.16)	0.33
Gender							
Male	412	61	Ref.		4	Ref.	
Female	53	80	0.72 (0.45–1.17)	0.187	9	0.87 (0.53–1.42)	0.572
Race							
Han	294	71	Ref.		4	Ref.	
Minority	171	52	1.12 (0.85–1.48)	0.434	6	0.95 (0.70–1.28)	0.723
BMI							
≤25	381	68	Ref.		5	Ref.	
>25	84	57	1.03 (0.74–1.44)	0.868	5	0.86 (0.59–1.25)	0.433
Smoking status							
None	304	73	Ref.		5	Ref.	
Ever	161	44	1.24 (0.93–1.64)	0.137	3	1.31 (0.96–1.78)	0.094
Drinking status							
None	286	75	Ref.		5	Ref.	
Ever	182	52	1.24 (0.94–1.62)	0.129	4	1.20 (0.89–1.63)	0.236
Child‐Pugh score							
A	393	65	Ref.		4	Ref.	
B	62	39	1.26 (0.86–1.85)	0.234	**5**	1.10 (0.70–1.74)	0.679
Cirrhosis							
No	58	82	Ref.		7	Ref.	
Yes	407	58	1.17 (0.77–1.80)	0.462	4	1.94 (1.13–3.32)	**0.016**
AFP							
≤400 (ng/ml)	238	63	Ref.		5	Ref.	
>400 (ng/ml)	194	42	1.31 (0.99–1.73)	0.063	3	1.09 (0.79–1.50)	0.061
NA	33						
BCLC stage							
A	275	96	Ref.		**7**	Ref.	
B/C	190	34	1.92 (1.35–2.73)	**<0.001**	**3**	1.46(1.08–1.98)	**0.014**
Antiviral therapy							
Yes	167	81	Ref.		4	Ref.	
No	298	52	0.76(0.55–1.04)	0.083	5	1.04 (0.76–1.40)	0.821
Adjuvant TACE							
Yes	265	48	Ref.		4	Ref.	
No	200	101	1.27 (0.96–1.69)	0.100	5	0.87 (0.62–1.22)	0.420
Radical resection							
Yes	261	74	Ref.		3	Ref.	
No	194	48	1.25 (0.95–1.64)	0.111	6	1.55 (1.14–2.11)	**0.005**
NA	10						
Pathological							
Grade							
Well	25	79	Ref.	0.658	5	Ref.	0.390
Moderately	359	57	1.30 (0.69–2.46)	0.423	5	1.34 (0.67–2.66)	0.408
Poorly	12	NA	1.02 (0.32–3.27)	0.969	1	2.31 (0.71–7.63)	0.171
NA	69						
Tumor size							
≤5 cm	151	123	Ref.		13	Ref.	
>5 cm	314	42	2.08 (1.50–2.90)	**<0.001**	3	1.68 (1.19–2.36)	**0.003**
No. of tumors							
Single (*n* = 1)	346	71	Ref.		5	Ref.	
Multiple (*n* > 1)	119	39	1.52 (1.14–2.03)	**0.005**	4	1.20 (0.86–1.68)	0.295
Regional invasion							
Absence	395	73	Ref.		6	Ref.	
Presence	70	40	1.72 (1.20–2.48)	**0.003**	2	1.87(1.29–2.70)	**0.001**
Intrahepatic metastasis							
Absence	253	82	Ref.		6	Ref.	
Presence	212	39	1.87 (1.43–2.46)	**<0.001**	**5**	0.73(0.54–0.98)	**0.034**
Vascular invasion							
Absence	383	80	Ref.		5	Ref.	
Presence	82	19	3.40 (2.50–4.62)	**<0.001**	2	1.60(1.12–2.28)	**0.010**
PVTT							
No	394	79	Ref.		5	Ref.	
Yes	71	18	3.22 (2.35–4.12)	**<0.001**	2	1.98(1.35–2.90)	**<0.001**

BMI, body mass index; AFP, alpha‐fetoprotein; TACE, transarterial chemoembolization; PVTT, portal vein tumor thrombus; BCLC stage, Barcelona Clinic Liver Cancer classification stage; OS, overall survival; MST, median survival time; RFS, recurrence‐free survival; MRT, median recurrence time; HR, hazard ratio; 95% CI, 95% confidence interval; Ref., reference.

Note: *HR and *P value for univariate survival analysis of Cox proportional hazard regression model.

This study was carried out under the approval of the Ethics Committee of the First Affiliated Hospital of Guangxi Medical University.

### SNP selection and genotyping

Tumor tissues and adjacent nontumor tissues were collected after hepatectomy, and immediately snap‐frozen and stored at −80°C. DNA extraction and purity met the experimental need. Four candidate Notch receptor genes (Notch1‐4) were assessed to estimate effects of their variants on survival in HBV‐related HCC patients after surgery. SNPs were selected via SNP selection tools (http://snpinfo.niehs.nih.gov/snpinfo/snpfunc.htm). The process and criteria for selection included minor allele frequency (MAF) of SNP >5% in the Asian population (CHB) in the HapMap database and location of functional SNPs in exons, miRNA binding sites of the 3′ untranslated region, the transcription factor binding site of the 5′ flanking regionand splice sites.

Based on the selection strategy, 19 SNPs in Notch pathway receptors were analyzed (Table 2), among which one SNP (rs1043996) in Notch3 and three SNPs (rs422951, rs520692, rs3830041) in Notch4 were selected for further analyses. Strict quality controls were carried out during genotyping, and 100% concordance in selected samples genotyped in duplicate.

**Table 2:** Association of Notch receptor SNPs with clinical outcomes of HCC patients

SNP	Chr	Position	Gene	Allele	Function	MAF	HWE	OS				RFS			
								*P*	HR (95% CI)	*P* a	HR^a^ (95%cl)	*P*	HR (95% CI)	*P* a	HR^a^ (95% CI)
rs4489420	9	139418260	NOTCH1	*Ma*	synonymous	0.1305	0.122	0.729	1.04 (0.83–1.32)	0.556	1.09 (0.81–1.47)	0.097	0.75 (0.53–1.05)	0.250	0.80 (0.55–1.17)
rs835574	1	120463230	NOTCH2	T/C	silent	0.1242	0.870	0.985	1.00 (0.78–1.30)	0.848	0.97 (0.70–1.34)	0.124	0.76 (0.54–1.08)	0.402	0.84 (0.57–1.26)
rs1043996	19	15295134	NOTCH3	A/G	synonymous	0.3991	0.0003	0.573	1.05 (0.88–1.25)	**0.023**	1.43 (1.05–1.93)	**0.047**	0.78 (0.61–0.99)	0.514	0.93 (0.73–1.17)
rs422951	6	32188383	NOTCH4	*TIC*	missense	0.1771	0.742	0.66	1.24 (0.97–1.56)	**0.042**	0.73 (0.54–0.99)	0.091	1.29 (0.96–1.73)	0.990	1.00 (0.73–1.36)
rs520692	6	32188640	NOTCH4	*TIC*	missense	0.1538	0.893	0.219	1.16 (0.91–1.48)	**0.028**	0.70 (0.51–0.96)	0.195	1.27 (0.90–1.67)	0.976	1.01 (0.74–1.37)
rs206018	6	32177880	NOTCH4	G/C	silent	0.2115	0.674	0.522	1.08 (0.85–1.36)	0.675	0.95 (0.75–1.21)	0.055	1.40 (0.99–1.96)	0.108	1.29 (0.95–1.77)
rs379464	6	32186348	NOTCH4	T/C	silent	0.1981	0.982	0.237	1.16 (0.91–1.48)	0.385	0.90 (0.70–1.15)	0.031	1.48 (1.04–2.12)	0.074	1.35 (0.97–1.87)
rs394657	6	32187023	NOTCH4	*Ala*	silent	0.1583	0.991	0.087	1.25 (0.97–1.60)	0.859	0.97 (0.72–1.31)	0.615	1.09 (0.78–1.51)	0.908	0.98 (0.71–1.36)
rs396960	6	32191581	NOTCH4	*AIT*	silent	0.3122	0.288	0.660	1.05 (0.86–1.27)	0.236	0.88 (0.71–1.09)	0.615	1.07 (0.83–1.37)	0.364	1.12 (0.88–1.42)
rs404860	6	32184345	NOTCH4	*TIC*	silent	0.3550	0.423	0.110	0.89 (0.73–1.03)	0.782	1.03 (0.84–1.27)	0.679	0.95 (0.74–1.22)	0.701	0.95 (0.75–1.21)
rs415929	6	32189032	NOTCH4	*TIC*	synonymous	0.1846	0.437	0.210	1.15 (0.93–1.42)	0.190	0.84 (0.64–1.09)	0.271	1.17 (0.89–1.54)	0.845	1.03 (0.78–1.36)
rs429853	6	32187202	NOTCH4	*TIC*	silent	0.1424	0.927	**0.033**	1.33 (1.02–1.76)	0.474	0.89 (0.65–1.22)	0.347	1.17 (0.84–1.63)	0.769	0.95 (0.68–1.34)
rs2071278	6	32165444	NOTCH4	*Ala*	silent	0.2635	0.563	0.160	1.16 (0.94–1.42)	0.654	1.06 (0.84–1.33)	0.256	1.17 (0.89–1.54)	0.102	1.25 (0.96–1.64)
rs2071285	6	32180431	NOTCH4	T/A	silent	0.1917	0.630	0.142	1.19 (0.94–1.51)	0.630	0.94 (0.72–1.22)	0.541	1.10 (0.81–1.49)	0.306	1.17 (0.87–1.57)
rs2071286	6	32179896	NOTCH4	T/C	silent	0.1040	0.118	0.804	1.04 (0.76–1.42)	0.967	0.99 (0.70–1.41)	0.845	0.96 (0.65–1.42)	0.975	1.01 (0.68–1.49)
rs2071287	6	32170433	NOTCH4	T/C	silent	0.4193	0.271	0.045	0.83 (0.69–0.99)	0.271	1.13 (0.91–1.42)	**0.047**	0.78 (0.61–1.00)	0.082	0.81 (0.63–1.03)
rs2854050	6	32185605	NOTCH4	*Ala*	silent	0.1906	0.670	0.154	1.18 (0.94–1.49)	0.542	0.92 (0.71–1.20)	0.562	1.10 (0.81–1.48)	0.331	1.16 (0.86–1.55)
rs3830041	6	32191339	NOTCH4	*TIC*	silent	0.0685	0.214	0.824	0.97 (0.64–1.42)	**0.001**	0.53 (0.37–0.75)	0.319	1.28 (0.79–2.10)	0.902	0.975 (0.65–1.4)
rs8192575	6	32166384	NOTCH4	C/G	silent	0.1929	0.570	0.140	0.84 (0.66–1.06)	0.499	1.09 (0.84–1.42)	0.464	0.89 (0.66–1.21)	0.343	0.87 (0.65–1.16)

SNP, single nucleotide polymorphism; Chr, chromosome; MAF, minor allele frequency; HWE, Hardy–Weinberg equilibrium; HR, hazard ratio; 95% CI,95% confidence interval.

a *P* adjustment for age, gender, BMI, race, smoking status, drinking status, Child‐Pugh class, cirrhosis, AFP level, BCLC stage, adjuvant antiviral therapy, adjuvant TACE, radical resection, pathological grade, intrahepatic metastasis, vascular invasion, regional invasion, and PVTT.

Note: bold value means that SNP is significantly associated with OS or RFS.

### Follow‐up

The follow‐up of enrolled patients was performed through hospital visits or telephone until death or the cut‐off date (September 30, 2014). The duration of follow‐up was defined as the number of months from the date of operation to death or cut‐off date.

### Analysis of Notch3 and Notch4 expression and polymorphisms

We collected twenty paired of tumor and adjacent nontumor tissues and immediately put into RNA fixer liquid (Aidlab) and stored in −80°C for RNA extraction from November 2016 to January 2017. Quantitative reverse transcription ‐ PCR was performed to calculate mRNA relative expression of Notch3 and Notch4 in 20 paired tissues. The expression of mRNAs relative to GAPDH gene expression was determined using the 2^−∆CT^ method. SNPs variants of Notch3 and Notch4 have sequenced. The primers are listed as follow:

Notch3, Forward primer, 5’‐TGTGAGACCGATGTCAACGAG‐3’, Reverse primer, 5’‐TGTGAAGCCTGCCATACAGATAC‐3’. Notch4, Forward primer, 5’‐AATGCGAGGAAGATACGGAGTG‐3’, Reverse primer, 5’‐GGACGGAGTAAGGCAAGGAG‐3’.

### Analysis of Notch receptor expression from the online database

To further analyze the association of mRNA expression of Notch receptors with survival of HBV‐related HCC patients, we exploited online data on HBV‐related HCC cohort GSE14520 from the Gene Expression Omnibus (GEO) database (http://www.ncbi.nlm.nih.gov/geo/). Further studies were performed on 221 patients from Fudan University (Shanghai, China). All patients met our inclusion criteria.

### Statistical analysis

Hardy–Weinberg equilibrium (HWE) for each SNP was estimated using a goodness‐of‐fit chi‐squared test with one degree of freedom. Linkage disequilibrium (LD) between SNPs in Notch receptor genes was analyzed using Gabriel's algorithm in Haploview version 4.2 23. Overall survival (OS) was defined from the date of surgery to the date of death or cut‐off. Median survival time (MST) and median recurrence time (MRT) were calculated via the Kaplan–Meier method with log‐rank test.

The Cox proportional hazards regression model was used to perform univariate and multivariate survival analyses. Hazard ratios (HR) and 95% confidence intervals (CI) were calculated after adjustment for clinicopathological parameters. The 75th percentile mRNA expression of Notch receptors in the total population was used as the cut‐off point to define lower and higher expression groups 24. Analysis of mRNA expression of Notch receptors in relation to clinical outcomes was performed using the Cox proportional hazards regression model. All statistical analyses were carried out by SPSS version 21.0 (IBM), with *P* values <0.05 considered significant.

## Results

### Patient characteristics and analysis of prognosis

The clinicopathological characteristics of all patients are summarized in Table 1. In total, 465 HBV‐related HCC patients with a median age of 45 years (range, 14–75 years) were enrolled. The duration of follow‐up was 3–125 months and MST was 32 months. The longest time of recurrence‐free survival was 96 months and MRT of patients subjected to radical resection was 5 months.

Univariate analysis showed that BCLC stages B and C (HR = 1.92, 95% CI = 1.35–2.73), tumor size >5 cm (HR = 2.08, 95% CI = 1.50–2.90), multiple tumors (*n* > 1) (HR = 1.52, 95% CI = 1.14–2.03), regional invasion (HR = 1.72, 95% CI = 1.20–2.48), intrahepatic metastasis (HR = 1.87, 95% CI = 1.43–2.46), vascular invasion (HR = 3.40, 95% CI = 2.50–4.62), and PVTT (HR = 3.22, 95% CI = 2.35–4.12) have significant negative effects on OS, compared to BCLC stage A, tumor size ≤ 5 cm, single tumor (*n* = 1), nonregional invasion, nonintrahepatic metastasis, nonvascular invasion, and non‐PVTT, respectively. Patients with cirrhosis (HR = 1.94, 95% CI = 1.13–3.32), BCLC stages B and C (HR = 1.46, 95% CI = 1.08–1.98), nonradical resection (HR = 1.55, 95% CI = 1.14–2.11), tumor size >5 cm (HR = 1.68, 95% CI = 1.19–2.36), regional invasion (HR = 1.87, 95% CI = 1.29–2.70), intrahepatic metastasis (HR = 0.73, 95% CI = 0.54–0.98), vascular invasion (HR = 1.60, 95% CI = 1.12–2.28), and PVTT (HR = 1.98, 95% CI = 1.35–2.90) were at higher risk of tumor recurrence, compared with noncirrhosis, BCLC stage A, radical resection, tumor size ≤ 5 cm, nonregional invasion, nonintrahepatic metastasis, nonvascular invasion, and non‐PVTT patient groups, respectively.

### Association of Notch receptor SNPs with clinicopathological characteristics

**Figure 1:**
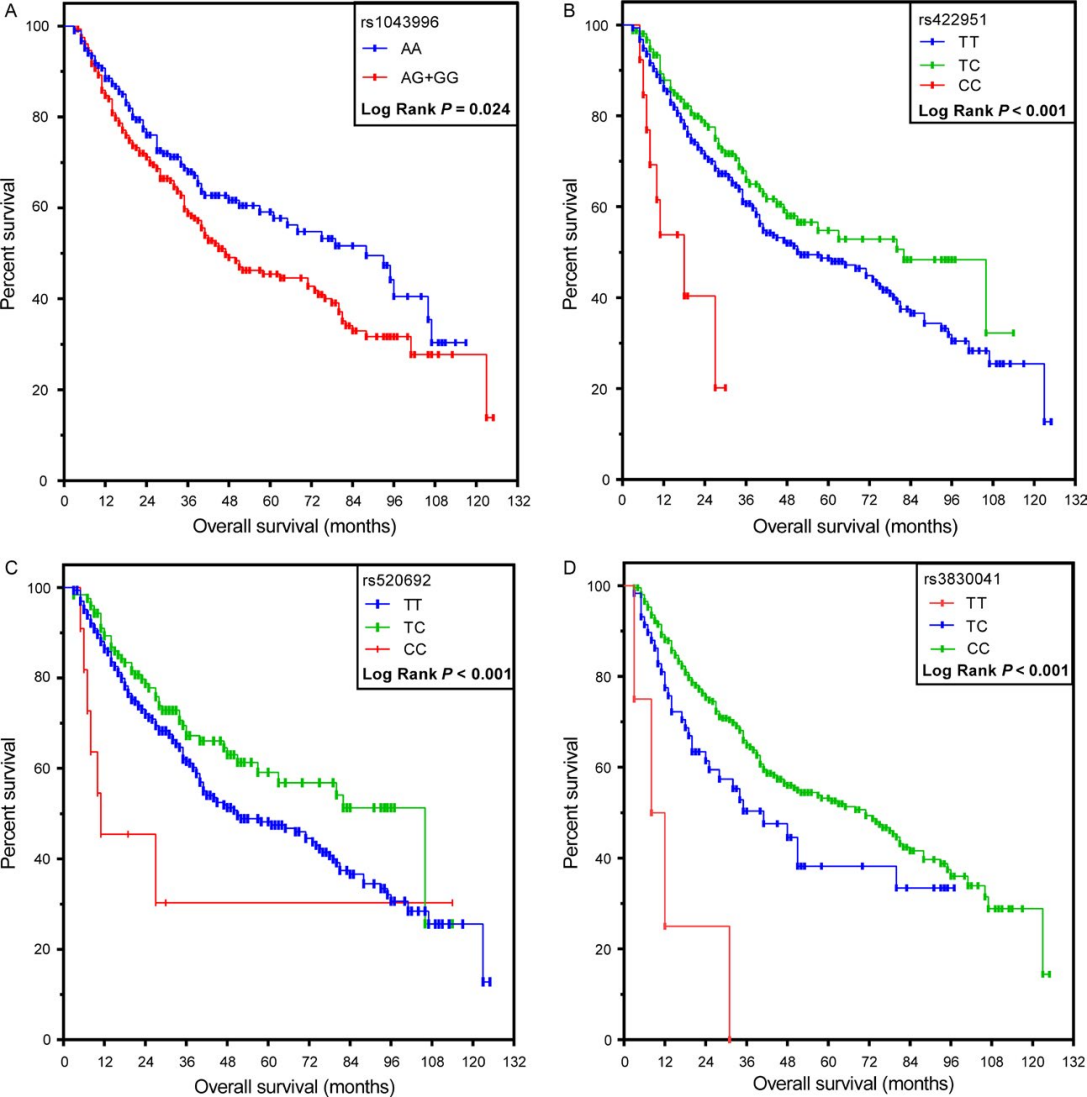
Kaplan–Meier plots of overall survival stratified by SNPs in clinical outcomes of HBV‐related hepatocellular carcinoma (HCC) patients. (A) rs1043996 (AA versus AG/GG), with AG and GG included in one group, (B) rs422951 (TT versus TC and CC), (C) rs520692 (TT versus TC and TT), (D) rs3830041 (TT versus TC and CC).

According to selection criteria, associations of 19 SNPs in four Notch receptors with OS and RFS in HCC patients were analyzed. rs1043996 in Notch3 and rs422951, rs520692 and rs3830041 in Notch4 were significantly associated with OS tested by the Cox regression model after adjustment for clinicopathological characteristics. However, none of SNPs were significantly associated with RFS (Table 2). The adjusted overall survival curve indicated that the Notch3 rs1043996 AA genotype has a positive effect on OS, compared with the AG/GG genotypes (*P *=* *0.024; HR = 1.43, 95% CI:1.05–1.93) (Table 3, Fig. 1). Moreover, genotypes TT and TC in rs422951 (*P *=* *0.004; HR = 3.53, 95% CI:1.50–8.32) and rs520692 (*P *=* *0.001; HR = 4.88, 95% CI: 1.89–12.59) exerted significant positive effects on OS, compared with the GG genotype, while patients with the rs3830041 TT genotype exhibited significantly poorer MST than those with TC/CC genotypes (*P *=* *0.002; HR = 0.19, 95% CI: 0.07–0.54) (Table 3, Fig. 1).

**Table 3:** Genotypes of Notch receptor SNPs in relation to overall survival of HCC patients

SNP	Patients (*n* = 465)	MST (months)	Overall survival			
			Crude HR (95% CI)	Crude *P*	AdjustedHR^a^ (95%CI)	Adjusted *P* a
rs1043996						
AA	184	88	Ref.	0.080	Ref.	0.075
AG	194	47	1.40 (1.04–1.90)	0.029	1.42 (1.03–1.97)	0.32
GG	87	50	1.33 (0.90–1.97)	0.148	1.43 (0.95–2.16)	0.091
AG+GG	281	47	1.38 (1.04–1.84)	0.026	1.43 (1.05–1.93)	0.024
rs422951						
TT	312	52	Ref.	0.005	Ref.	<0.001
TC	139	106	0.70 (0.51–0.96)	0.026	0.57 (0.39–0.83)	0.004
CC	14	18	2.18 (1.07–4.46)	0.033	2.97 (1.25–7.05)	0.014
TT+TC	451	65	2.40 (1.18–4.90)	0.016	3.53 (1.50–8.32)	0.004
rs520692						
TT	330	51	Ref.	0.005	Ref.	<0.001
TC	124	106	0.70 (0.50–0.97)	0.032	0.55 (0.37–0.80)	0.002
CC	11	11	2.37 (1.11–5.07)	0.026	3.37 (1.29–8.82)	0.013
TT+TC	454	65	2.58 (1.21–5.52)	0.014	4.88 (1.89–12.59)	0.001
rs3830041						
TT	4	8	Ref.	<0.001	Ref.	<0.001
TC	59	41	0.21 (0.07–0.59)	0.003	0.18 (0.06–0.54)	0.002
CC	402	71	0.14 (0.05–0.38)	<0.001	0.11 (0.04–0.31)	<0.001
TC+CC	461	65	0.15 (0.06–0.40)	<0.001	0.19 (0.07–0.54)	0.002

HR, hazard ratio; 95% CI, 95% confidence interval, MST, median survival time.

a *P* adjustment for age, gender, BMI, race, smoking status, drinking status, Child‐Pugh class, cirrhosis, AFP level, BCLC stage, adjuvant antiviral therapy, adjuvant TACE, radical resection, pathological grade, intrahepatic metastasis, vascular invasion, regional invasion, and PVTT.

### Linkage disequilibrium and Haplotype analysis

**Figure 2:**
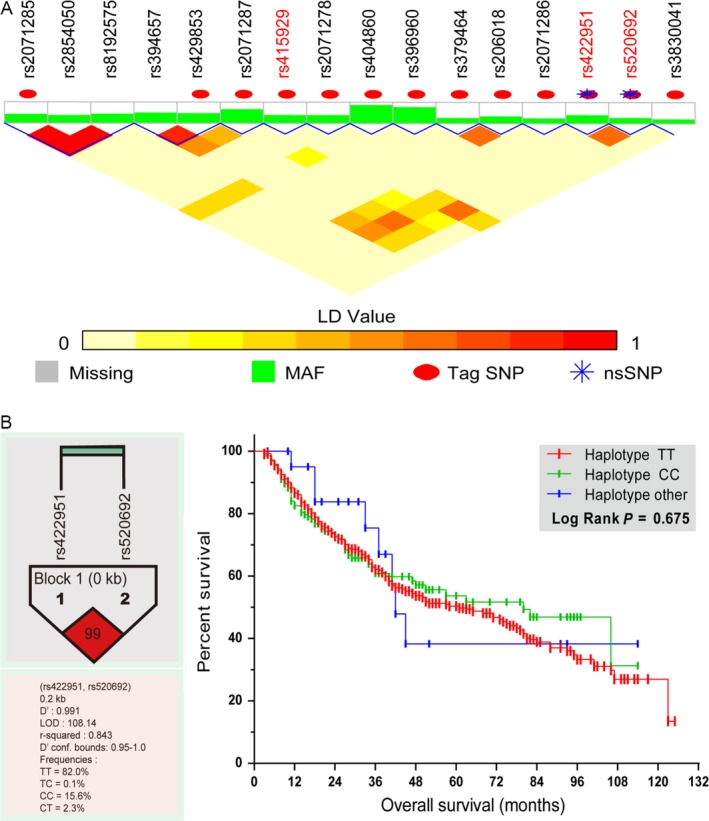
Linkage disequilibrium (LD) and haplotype analysis of 19 SNPs. (A) Five haplotype blocks were identified: (rs2071285, rs2854050 and rs8192575), (rs394657, and rs429853), (rs429853 and rs2071287), (rs379464 and rs206018), and (rs422951 and rs520692). rs422951 and rs520692 were both tag and nonsynonymous SNPs. (B) Haploview LDgraph of rs422951 and rs520692 visualized using Haploview software 4.2. rs422951 was in strong LD with rs520692, r^2^ = 0.843.

We analyzed Linkage disequilibrium (LD) and haplotyes for the 19 SNPs (Fig. 2) using the SNP analysis tool (http://snpinfo.niehs.nih.gov/snpinfo/snpfunc.htm). Five haplotype blocks were identified in Notch4: (rs2071285, rs2854050, and rs8192575), (rs394657 and rs429853), (rs429853 and rs2071287), (rs379464 and rs206018), and (rs422951 and rs520692). In particular, the haplotype block of rs422951 in strong LD with rs520692 (r^2^ = 0.843) was significantly associated with OS, and both were tagged and nonsynonymous SNPs.

### Stratified analysis of four valuable Notch SNPs in association with clinicopathological factors

**Figure 3:**
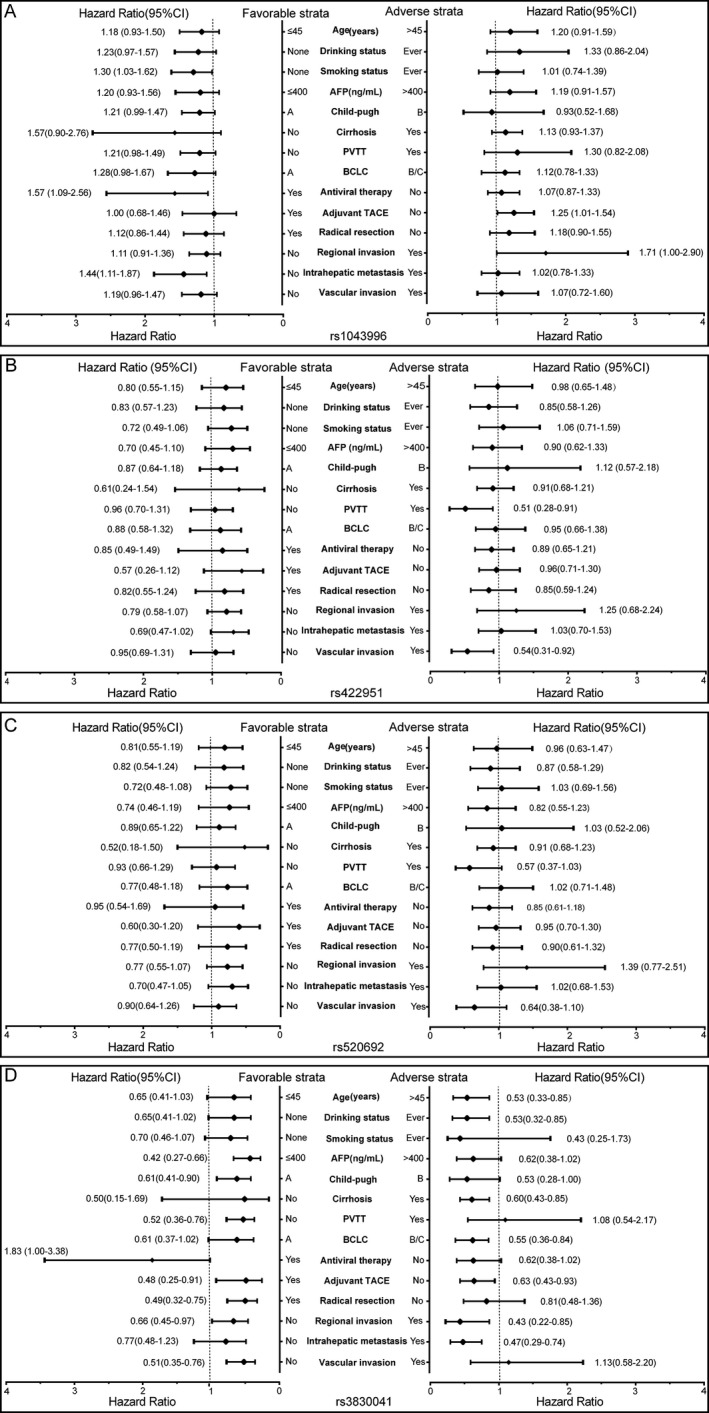
Stratification analysis of the association of rs1043996, rs422951, rs520692, rs3830041 with overall survival in HBV‐related HCC patients. Stratification was based on favorable and adverse strata.

Four Notch receptor SNPs (rs1043996, rs422951, rs520692, rs3830041) showing significant association with clinical outcomes were further analyzed. Stratified analysis by favorable and adverse strata was performed between the genotypes of the four SNPs and clinicopathological characteristics of patients (Fig. 3). Patients carrying rs1043996 AG/GG genotypes displayed increased risk of death in the subgroups of age >45 years, drinking status, noncirrhosis, PVTT, antiviral therapy, no adjuvant TACE, and regional invasion. The rs422951 CC genotype was associated with increased risk of death in the following subgroups: age >45 years, smoking status, AFP >400 ng/mL, non‐PVTT, Child‐Pugh B, cirrhosis, BCLC stages B and C, nonadjuvant TACE, regional invasion, and intrahepatic metastasis. Similar results were observed for rs520692 with the CC genotype. The TT variant of rs3830041 was associated with increased death risk in the following subgroups: age ≤45 years, nonsmoking status, AFP >400 ng/ml, cirrhosis, PVTT, antiviral therapy, nonadjuvant TACE, nonradical resection, nonregional invasion, nonintrahepatic metastasis, and vascular invasion.

### Association of Notch receptors expression and polymorphisms

**Figure 4:**
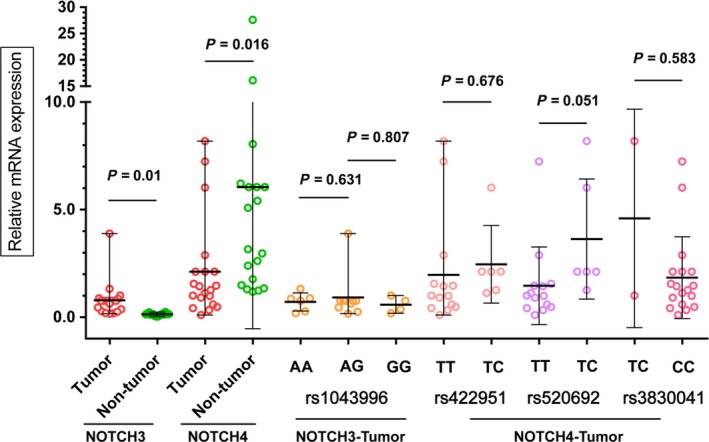
Relative mRNA Expression of Notch3 and Notch4 in 20 paired tissues and association of Notch3 and Notch4 SNPs and mRNA Relative Expression in 20 tumor samples. The mRNA Expression is relative to GAPDH gene expression.

Our results showed that the relative mRNA expression of Notch3 tumor samples is significantly higher than paired nontumor samples. The results of Notch4 are just reversed (Fig. 4). And our study did not present that polymorphisms significantly affected the expression of Notch receptors. But our data demonstrated that the variant types of rs520692 obviously affected the mRNA expression of Notch4.

### Association of Notch receptors expression with clinical outcomes in HBV‐related HCC patients

**Figure 5:**
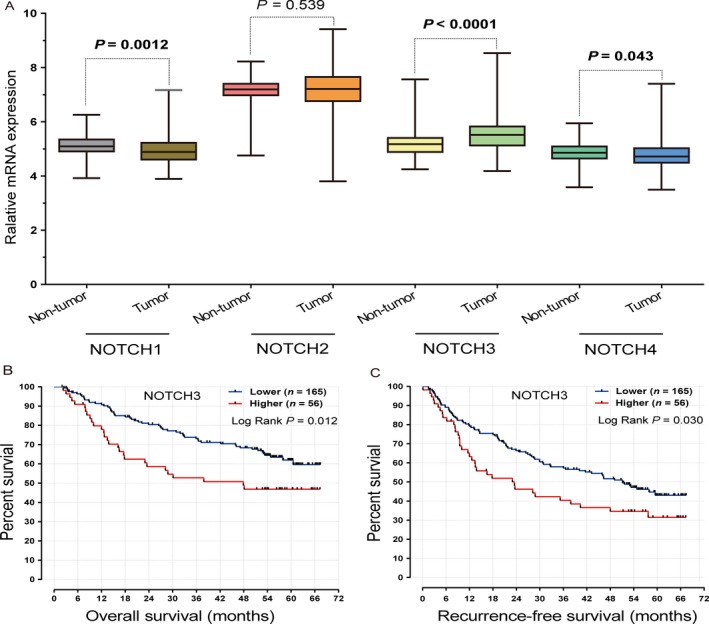
mRNA expression of Notch receptor genes in HCC tissues and adjacent normal tissues, and association with OS and RFS. (A) Notch1, Notch3, and Notch4 expression levels were significantly different between HCC tissues and nontumor normal tissues. (B) and (C) Kaplan–Meier plot of clinical outcomes of HCC patients in relation to Notch3 mRNA expression. The 75th percentile of mRNA expression of Notch3 was used as the cut‐off point to define lower and higher expression groups.

We additionally retrieved data on the HBV‐related HCC cohort GSE14520 from the GEO database (http://www.ncbi.nlm.nih.gov/geo/). Overall, 221 patients were included to further assess the effects of Notch receptor expression on clinical outcomes of HBV‐related HCC. Compared with nontumor normal tissues, downregulation of Notch1 and Notch4 was observed in HCC tissues, while Notch2 expression was not significantly different between tumor and normal tissues. In particular, Notch3 expression was markedly upregulated in HCC tissues (*P *<* *0.0001) (Fig. 5A). Further examination of the association of Notch3 expression with clinical outcomes revealed that expression of Notch3 is significantly associated with OS and RFS (Table 4, Fig. 5B,C). Specifically, patients with lower Notch3 expression showed better OS and RFS than those with higher Notch3 expression (*P *=* *0.002, HR* *=* *2.11, 95% CI* *=* *1.32–3.37 and *P *=* *0.001, HR* *=* *1.96, 95% CI* *=* *1.31–2.93, respectively).

**Table 4:** Association of Notch mRNA expression with clinical outcomes of HCC

Gene	Patients (*n* = 221)	Overall survival			Recurrence‐free survival		
		MST (months)	HR^a^ (95%CI)	*P* a	MRT (months)	HR^a^ (95%CI)	*P* a
NOTCH1							
Lower	165	NA	Ref.		45	Ref.	
Higher	56	NA	1.18 (0.71–1.93)	0.524	46	1.04 (0.68–1.60)	0.846
NOTCH2							
Lower	165	NA	Ref.		36	Ref.	
Higher	56	NA	1.42 (0.83–2.42)	0.203	59	0.71 (0.46–1.10)	0.128
NOTCH3							
Lower	165	NA	Ref.		51	Ref.	
Higher	56	47.9	2.11 (1.32–3.37)	**0.002**	23	1.96 (1.31–2.93)	**0.001**
NOTCH4							
Lower	165	NA	Ref.		46	Ref.	
Higher	56	57.9	1.35 (0.85–2.15)	0.201	32	1.20 (0.81–1.79)	0.370

MST, median survival time; MRT, median recurrence time; HR, hazard ratio; 95% CI, 95% confidence interval; Ref., reference.

The 75^th^ percentile of mRNA expression in the total population was used as the cut‐off point to define low and high expression groups.

a Adjustment for age, gender, cirrhosis, BCLC stage, serum AFP level.

Bold values represent that notch3 mRNA expression was significantly associated with overall survival and recurrence‐free survival respectively adjusted for age, gender, cirrhosis, BCLC stage, serum AFP level.

## Discussion

In this study, we investigated the effects of SNPs in Notch receptors (Notch1‐4) on prognosis of 465 HBV‐related HCC patients subjected to operation. Our research disclosed that rs1043996 in Notch3 and rs422951, rs520692 and rs3830041 in Notch4 contribute significantly to OS, but show no significant association with RFS. This is the first study to highlight associations of SNPs in Notch receptors with clinical outcomes of HBV‐related HCC.

A number of reports have demonstrated that SNPs in Notch receptors are linked to risk and prognosis of several diseases. For instance, SNPs in Notch1 and Notch2 are associated with risk of breast carcinoma 25, 26. Genetic variants of Notch3 and Notch4 genes are associated with cerebral small vessel disease 27 and Alzheimer's Disease 28, respectively. However, no relationship between variants of Notch receptors and outcomes of HCC patients has been documented until now. Data from the present study indicate that rs1043996, rs422951, rs520692, and rs3830041 are significantly associated with risk of death in HCC patients. rs1043996 with the AA variant is a protective genotype that contributes significantly to longer overall survival, compared to the AG/GG genotypes. rs422951 and rs520692 with the CC genotype are variant alleles that negatively affect overall survival relative to the TT/TC genotypes. Conversely, patients carrying the rs3830041 AA genotype in Notch4 had significantly shorter MST, compared to those carrying AA/AG. These findings suggest that the G allele of rs1043996, C allele of rs422951 and rs520692 and T allele of rs3830041 are potential risk variants for HBV‐related HCC, compared with the A allele of rs1043996, T allele of rs422951 and rs520692 and C allele of rs3830041, respectively.

Tag SNPs are a small number of SNPs sufficient to capture most of the haplotpye structure of the human genome with minimal errors and have good predictive ability 23, 29. LD is one of main approaches used for analysis of haplotype block structures 23, 30.Nonsynonymous SNPs (nsSNP) are coding mutations that introduce amino acid alterations leading to changes in protein sequences. Consequently, nsSNPs often induce changes in protein function 31 and cause disease or susceptibility to diseases, including breast cancer 32, gastric cancer 33, and HCC 34. We found that the haplotype block of rs422951 is in strong LD with rs520692. The two polymorphisms in this block were both tag and nonsynonymous SNPs and significantly associated with OS.

Earlier researches have clearly indicated that Notch receptor expression has association with outcomes in HCC patients. Ahn et al. 35 reported that Notch1 expression can be effectively used to predict shorter disease‐free and disease‐specific survival in HCC patients after surgery and Notch4 overexpression can be used to predict shorter disease‐specific survival. Accumulating evidence 36 has revealed an association of high Notch3 expression with poor prognosis of HCC patients. Here, we analyzed data obtained from the GEO database to identify potential associations between mRNA expression of Notch pathway genes and clinical outcomes of HBV‐related HCC patients. We observed significant differences in Notch1, Notch3, and Notch4 expression between tumor and adjacent normal tissues, but not Notch2. Moreover, Notch1, Notch2, and Notch4 expression were not associated with OS and RFS. Importantly, Notch3 expression was higher in tumor tissues compared with surrounding tissues and led to poorer OS and RFS outcomes, although the mechanisms underlying this association remain to be identified. Previous experiments 37 have disclosed that the Notch signaling pathway suppresses HCC cell invasion via ERK1/2‐mediated downregulation MMP‐2, MMP‐9, and VEGF. Another study 38 showed that Notch3 suppresses p53 expression in HCC and Notch3‐silenced cells regulate p53 protein expression at the post‐transcriptional level through Ciclin G1. Zhang et al. 39 demonstrated that Notch3 functions in regulating the stemness of HCC cells by interacting with the Wnt/*β*‐catenin pathway.

In summary, four SNPs (rs1043996 in Notch3, rs422951, rs520692, and rs3830041 in Notch4) appear to have significant association with OS in HBV‐related HCC patients. In particular, the A allele of rs1043996 and T allele of rs422951and rs520692 represent favorable genotypes, while the T allele of rs3830041 is possibly an unfavorable predictor of survival. Our data additionally provide evidence that rs422951 is in strong LD with rs520692 and both belong to tag SNP and nonsynonymous SNP groups. Moreover, Notch3 expression may be an effective prognostic predictor in HCC. The collective findings provide a new perspective for prediction and therapeutic strategies for HCC.

While significant results have been obtained in the present study, a number of limitations must be considered. The mechanisms underlying the association of the above SNPs of Notch pathway receptors with clinical outcomes in HCC remain to be established. In particular, rs422951 and rs520692 were both tag and nonsynonymous SNPs in strong LD, it is not known whether they lead to changes in protein sequences and functions. But our study shows that the variant types of rs520692 probably affected the mRNA expression of Notch4. Additionally, our results showed partial discrepancies with previous investigations on Notch pathway receptor expression in tumor tissues and their associations with clinical outcome. Further multicenter studies on larger patient populations are essential to obtain more accurate evidence on the prognostic value of Notch receptors in hepatocellular carcinoma.

## Conclusions

Four Notch SNPs (rs1043996 in Notch3, rs422951, rs520692, and rs3830041 in Notch4) are potential prognostic biomarkers for patients with HBV‐related HCC. Moreover, Notch3 expression may be a valuable prognostic predictor for HBV‐related HCC patients following hepatectomy.

## Conflicts of Interest

There is no conflict of interest disclosed in this study.
